# Mice with lung airway ciliopathy develop persistent *Mycobacterium abscessus* lung infection and have a proinflammatory lung phenotype associated with decreased T regulatory cells

**DOI:** 10.3389/fimmu.2022.1017540

**Published:** 2022-11-25

**Authors:** Audrey Nava, Andrew C. Hahn, Terry H. Wu, Thomas F. Byrd

**Affiliations:** ^1^ Center for Infectious Disease and Immunity, The University of New Mexico Health Science Center, Albuquerque, NM, United States; ^2^ Department of Medicine, The University of New Mexico School of Medicine, Albuquerque, NM, United States

**Keywords:** nontuberculous mycobacteria, cilia, bronchiectasis, IFT88, CD4^+^IL17^+^, CD4^+^FoxP3^+^, interleukin 22, interleukin 6

## Abstract

**Introduction:**

Human pulmonary infection with non-tuberculous mycobacteria (NTM) such as *Mycobacterium abscessus* (Mabs) occurs in seemingly immunocompetent patients with underlying structural lung disease such as bronchiectasis in which normal ciliary function is perturbed. In addition to alterations in mucociliary clearance, the local immunologic milieu may be altered in patients with structural lung disease, but the nature of these changes and how they relate to NTM persistence remain unclear.

**Methods:**

We used a mouse strain containing a conditional floxed allele of the gene *IFT88*, which encodes for the protein Polaris. Deletion of this gene in adult mice reportedly leads to loss of cilia on lung airway epithelium and to the development of bronchiectasis. In a series of experiments, *IFT88* control mice and *IFT88* KO mice received different preparations of *Mabs* lung inocula with lung CFU assessed out to approximately 8 weeks post-infection. In addition, cytokine levels in bronchoalveolar lavage (BAL) fluid, lung T cell subset analysis, and lung histopathology and morphometry were performed at various time points.

**Results:**

*Mabs* embedded in agarose beads persisted in the lungs of *IFT88* KO mice out to approximately 8 weeks (54 days), while *Mabs* agarose beads in the lungs of *IFT88* control mice was cleared from the lungs of all mice at this time point. T cells subset analysis showed a decrease in the percentage of CD4^+^FoxP3^+^ T cells in the total lymphocyte population in the lungs of *IFT88* KO mice relative to *IFT88* control mice. Proinflammatory cytokines were elevated in the BAL fluid from infected *IFT88* KO mice compared to infected *IFT88* control mice, and histopathology showed an increased inflammatory response and greater numbers of granulomas in the lungs of infected *IFT88* KO mice compared to the lungs of infected *IFT88* control mice. Scanning lung morphometry did not show a significant difference comparing lung airway area and lung airway perimeter between *IFT88* KO mice and *IFT88* control mice.

**Discussion:**

Persistent lung infection in our model was established using *Mabs* embedded in agarose beads. The utility of using *IFT88* mice is that a significant difference in *Mabs* lung CFU is observed comparing *IFT88* KO mice to *IFT88* control mice thus allowing for studies assessing the mechanism(s) of *Mabs* lung persistence. Our finding of minimal differences in lung airway area and lung airway diameter comparing *IFT88* KO mice to *IFT88* control mice suggests that the development of a proinflammatory lung phenotype in *IFT88* KO mice contributes to *Mabs* lung persistence independent of bronchiectasis. The contribution of cilia to immune regulation is increasingly recognized, and our results suggest that ciliopathy associated with structural lung disease may play a role in NTM pulmonary infection via alteration of the local immunologic lung milieu.

## Introduction

*Mycobacterium abscessus* (*Mabs*) is an important emerging pathogen that causes treatment-refractory, progressive lung disease in patients with abnormal airways characterized by bronchiectasis (1–4). Persistent *Mabs* lung infection occurs in immunodeficient mice and in one study with immunocompetent mice using an agar bead lung inoculum (5–10). Although studies with immunodeficient mice have yielded important information regarding the role that host immune responses play in preventing infections, these models do not mimic persistent human pulmonary infection, which typically occurs in immunocompetent individuals who have lung airway disease. The importance of lung airway ciliopathy as a predisposing factor for development of progressive lung infection in immunocompetent humans is demonstrated in individuals who have primary ciliary dyskinesia. Affected individuals have alterations of genes involving proteins in the outer and/or inner dynein arms that give cilia and flagella their motility. These individuals develop bronchiectasis and infection with either *Mabs* or *MAC* as a clinical feature of primary ciliary dyskinesia. Predisposition to lung infection is not felt to involve systemic immunodeficiency (11).

To examine the role that abnormal lung airways play in persistent *Mabs* lung infection, we used the Cre/*loxP* system to delete the gene *IFT88* in mice. *IFT88* is an intraflagellar transport protein necessary for normal development of cilia. Mice that have lost the *IFT88* gene rapidly lose cilia and have been reported to develop bronchiectasis (12). We show that *Mabs* introduced into the lungs of mice as a low inoculum embedded in agarose beads persists for a prolonged period (at least 8 weeks) in mice that have lost *IFT88* (*IFT88* KO mice), while control mice clear lung infection. *Mabs*-infected *IFT88* KO mice have a pro-inflammatory cytokine profile compared to control mice with increased levels of IL-22, IL-6, IL-17, TNFα, and IL-1α in bronchoalveolar lavage (BAL) fluid. In addition, uninfected *IFT88* KO mice have significantly increased levels of IL-22 relative to uninfected control mice. Consistent with this data, we show that in both infected and uninfected mice there is a significant decrease in the percentage of CD4^+^FoxP3^+^ cells (T regs) in *IFT88* KO mice compared to control mice. Taken together, our results suggest that Th17/Treg imbalance in mice lacking lung airway cilia contributes to *Mabs* persistence in the lungs of *IFT88* KO mice.

## Methods

### Mice

The mouse model used in these studies was chosen based on a prior report that deletion of the gene *IFT88* using the Cre/*loxP* system lead to *de novo* development of bronchiectasis in adult mice without evidence of antecedent infection. The model utilizes a conditional mouse mutant for the gene *IFT88* that encodes for a protein playing an important role in development of normal cilia. In *Cre recombinase* positive mice homozygous for the *IFT88* floxed allele, treatment with tamoxifen reportedly lead to progressive development of bronchiectasis starting at 3 weeks following gene deletion with an approximate 80% reduction in IFT88 protein expression after 2-3 weeks. In contrast, mice with the *IFT88* floxed allele lacking *Cre recombinase* had wild type levels of IFT88 protein after tamoxifen administration (12). For our studies, deletion of *IFT88* was accomplished by breeding *IFT88* floxed mice (B6.129P2- Ift88^tm1Bky^/J, Jackson Laboratory; Bar Harbor, ME) with mice possessing a tamoxifen-inducible Cre recombinase expressed from the actin promoter (B6.Cg-Tg(CAG-Cre/Esr1*)5Amc/J, Jackson Laboratory). Approximately 28 days after birth, mice were genotyped (Transnetyx; Cordova, TN) for the floxed *Ift88* allele (forward primer 5’-GCGGCTGCAGAGATCCA-3’; reverse primer 5’-GGTATTGTTAGGAAGTAGTAAAACATAA-3’), the wildtype *Ift88* allele (forward primer 5’-AGTAAGCGGCTGCAGAGATC-3’; reverse primer 5’-AATTCTGGCTCTGAACACAATCC-3’) and *Cre* (forward primer 5’-TTAATCCATATTGGCAGAACGAAAACG-3’; reverse primer 5’-CAGGCTAAGTGCCTTCTCTACA-3’). After selection of *Cre recombinase* positive/*IFT88* floxed mice (experimental group) and *Cre recombinase* negative/*IFT88* floxed mice (control group), both experimental and control mice received tamoxifen (Sigma-Aldrich; St Louis, MO) dissolved in corn oil (2 mg/100 µl) as daily intraperitoneal injections for 5 days. Since both *IFT88* control and *IFT88* KO mice received tamoxifen, differences observed comparing these groups are not attributable to tamoxifen per se. In addition the short duration of tamoxifen treatment (5 days) relative to the total duration of the experiments (approximately 3 months after tamoxifen administration) argues against an ongoing effect in our experiments. Depending on the experiment, *Mabs* infection of mice occurred 6-12 weeks after completion of the tamoxifen treatment. In addition to bronchiectasis, the other structural abnormality previously noted in these mice after deletion of *IFT88* is the development of renal cysts 6 months after induction of the Cre/*lox* system (13), which is beyond the time frame of our experiments. It has also been found that loss of *IFT88* in mice results in alteration in feeding behavior leading to weight gain – this is felt to be due to loss of cilia in the hypothalamus involved in regulating appetite (14). We observed this effect in the *Cre recombinase* positive *IFT88* floxed mice after receiving tamoxifen. Observation of daily food intake by age-matched *Cre recombinase* negative *IFT88* floxed male and female mice for a week indicated that these mice ate approximately 2.0 grams of food (Taklad 2920X; Envigo; Indianapolis, IN) per day. Thus, all mice received this amount of food throughout the course of experiments to maintain stable weight.

The experimental protocol used in these studies was approved by Institutional Animal Care and Use Committee of the University of New Mexico Health Sciences Center.

### Bacteria and lung infection

The *Mabs* 390R rough strain used in this study has been previously described (5, 15–19). It has been determined to be *Mycobacterium abscessus* subspecies *abscessus* (UT Health, The University of Texas Health Sciences Center at Tyler, Barbara A. Brown-Eliot).

Working stocks were prepared by culturing 390R in Middlebrook 7H9 medium (Sigma-Aldrich; St Louis, MO) supplemented with oleic acid-albumin-dextrose-catalase (OADC; Sigma-Aldrich) and 0.5% Tween 80 (Sigma-Aldrich) for 25 ± 5 h at 37°C and 100 rpm. The culture was centrifuged for 5 min at 15,000*g* and 4°C and the supernatant was discarded. The pellet was re-suspended in 10 mL of PBS with 0.5% Tween 80. Large clumps were allowed settle for 20 min, and then the supernatant was transferred into a new tube with 10 to 15 0.5 mm glass beads (BioSpec Products; Bartlesville, OK) and vortexed vigorously. Aliquots of the resulting single cell suspension was flash frozen in a dry ice-ethanol bath and stored at -80°C. The concentration of the working stocks was determined by plating serial dilutions on Middlebrook 7H11 Agarose (Sigma-Aldrich).

Bacteria for lung inoculation were prepared using several different methods. For intranasal infection with a single cell suspension, the inoculum was prepared by serial dilution from the frozen working stock with DPBS. 50 μL of the inoculum was placed on the nares of lightly anesthetized mice and allowed to be inhaled. To compare intratracheal (i.t.) infection with single cell suspension and bacterial aggregates, a fresh culture was prepared and processed using a procedure similar to that described to produce the working stock. The culture after 28.5 h incubation contained a mixture of individual bacteria and aggregates and was delivered using a 22-gauge intravenous (i.v.) catheter (Terumo; Tokyo, Japan). The single cell suspension after removing large clumps and homogenizing with glass beads was delivered using a MicroSprayer aerosolizer (Penn-Century, Inc; Wyndmoor, PA). For both methods of i.t. delivery, mice were lightly anesthetized with isoflurane and placed onto a dosing platform. A small animal laryngoscope (Penn-Century, Inc) was used to visualize the epiglottis and aid in proper placement of the catheter and the microsprayer, and 50 μl of the inoculum was pushed into the lungs. For infection using *Mabs* embedded in agarose bead, agarose beads were prepared following the method previously described for preparation of *Pseudomonas aeruginosa*-embedded agarose beads with slight modifications (20). Briefly, 390R was cultured in enriched Middlebrook 7H9 medium for 26 to 27 h at 37° C. A single cell suspension was prepared in DPBS with 0.5% Tween 80. 1 mL of the suspension was mixed with 10 mL of sterile 2% (w/v) Middlebrook 7H11 agarose (Sigma-Aldrich) maintained at 48°C. The mixture was slowly poured into a 250 mL Erlenmeyer flask containing 200 mL of sterile mineral oil (Sigma-Aldrich) heated to 48°C and stirred at 500 rpm with magnetic stir bar. After 5 min, ice was added around the flask of oil to quickly cool the agarose down and the mixture was stirred at a slower speed for another 20 minutes. The beads were washed 4 to 5 times with DPBS, each time followed by a 20 min centrifugation at 16,274*g* to remove remaining mineral oil. The agarose beads were delivered using a 22-guage i.v. catheter as previously described.

### *Mabs* lung CFU

At indicated time points, mice were euthanized by intraperitoneal (i.p.) injection of 5.9 mg sodium pentobarbital (Vortech Pharmaceuticals; Dearborn, MI) in 100 μL. Lung tissues were removed aseptically and homogenized in 1 ml of DPBS using a BeadBeater (Biospec Products). The lung homogenates were serially diluted in DPBS, and then 10 μL of each dilution were spotted onto 7H11 agarose plates. After incubation at 37°C for 4 days, colonies were counted under a dissecting microscope.

### Lung histopathology and morphometry

The left lung was inflated using a lung inflation apparatus filled with 10% neutral buffer formalin. After 24 h, the trachea was tied off and the tissue was placed in 70% ethanol. The tissues were trimmed and processed by routine methods and stained with hematoxylin and eosin. In initial experiments, a veterinary pathologist examined the slides and scored them for the presence of cilia, inflammation and Clara cell hyperplasia. In a subsequent experiment, the veterinary pathologist examined H&E stained slides and scored them for the presence of granulomas, inflammation and histiocytes (nonlymphoid mononuclear cells). The HALO morphometry system (Indica Labs; Albuquerque, NM) was also used to quantify the extent of inflammation with the system trained to recognize areas of heavy inflammatory cell infiltration. In addition, the HALO morphometry system was used to quantify lung airway size by assessing airway perimeter and area.

### T cell subset analysis

Mice were injected i.p. with 150 U of heparin and euthanized 10 min later by i.p. injection of sodium pentobarbital. The lungs were then immediately removed with the trachea, bronchus, heart and vasculature, with or without lavage, and then perfused with 10 mL DPBS. The lung lobes were cut into small pieces and enzymatically digested with 30 µg/mL DNAse I (Sigma-Aldrich) and 0.7 mg/mL collagenase Type IV (Sigma-Aldrich) in RPMI 1640 medium for 1 hour at 37°C. The digested tissues were pushed through a 70 µm cell strainer (Corning; Tewksbury, MA) and a nylon wool column (G. Kisker, DE). Red blood cells were lysed in 1 mL RBC Lysis Buffer (ThermoFischer; Waltham, MA) at room temperature for 5 min, washed with HBSS, and resuspended in 1 mL of RPMI 1640 medium with 5% fetal calf serum. The cell suspension was layered over 3 mL of 30% Percoll in PBS (GE Healthcare, Cranbury, NJ) and centrifuged at 800g for 20 min to remove cell debris. Cells were analyzed without any antigen restimulation. Cells were incubated with monensin for 4 hours, fixed, surface stained with CD4-PerCP-Cy5.5 (clone RM4-5), permeabilized and stained using an antibody cocktail for IFNγ-FITC (clone XMG1.2), IL-17-PE (TC11-18H10.1), and FoxP3-Alex647 (clone MF23) or a cocktail of the respective isotype controls (antibodies, fixation, permeabilization solutions from BD Biosciences). Flow cytometry data was acquired on a C6 Plus Accuri (BD Biosciences) cytometer and analyzed with FlowJo (Treestar, Ashland OR). Singlet and lymphocyte gates were used with dead cells and debris excluded. Trypan blue staining was performed on all cell preparations analyzed by the flow cytometer. Our gating strategy is shown in the **Supplemental Figure**.

### BAL cytokine determination

Mice were euthanized by i.p. injection of sodium pentobarbital. The lungs were then immediately removed and a cannula was inserted into the trachea. The lungs were lavaged three times with 0.70 mL cold DPBS, and the bronchoalveolar lavage fluid (BAL) was pooled, frozen in a dry ice-ethanol bath and stored for subsequent cytokine measurements. Cytokine and chemokines were detected and quantified using magnetic beads coated with analyte-specific capture antibodies (R&D Systems; Minneapolis, MN) and BioPlex 200 Instrument (Bio-Rad; Hercules, CA).

### Statistics

For comparisons unpaired, two tailed test was performed using GraphPad Prism version 9.3.1 for Windows, GraphPad Software, San Diego, California USA, www.graphpad.com. If less than 3 values were available from one or both groups to be compared, statistical analysis was not performed.

## Results

### *Mabs* persists in the lungs of *IFT88* KO mice

*Mabs* can persist in the lungs of immunocompromised mice, and recent studies provide evidence that with a high titer infecting lung inoculum, and embedding the infecting inoculum in substrate the barriers to infection in the lungs of mice with normal lung airways can be overcome resulting in varying degrees of persistent infection (10, 21). There are no prior reports of *Mabs* pulmonary infection in mice with structurally abnormal lung airways.

**Figure 1:**
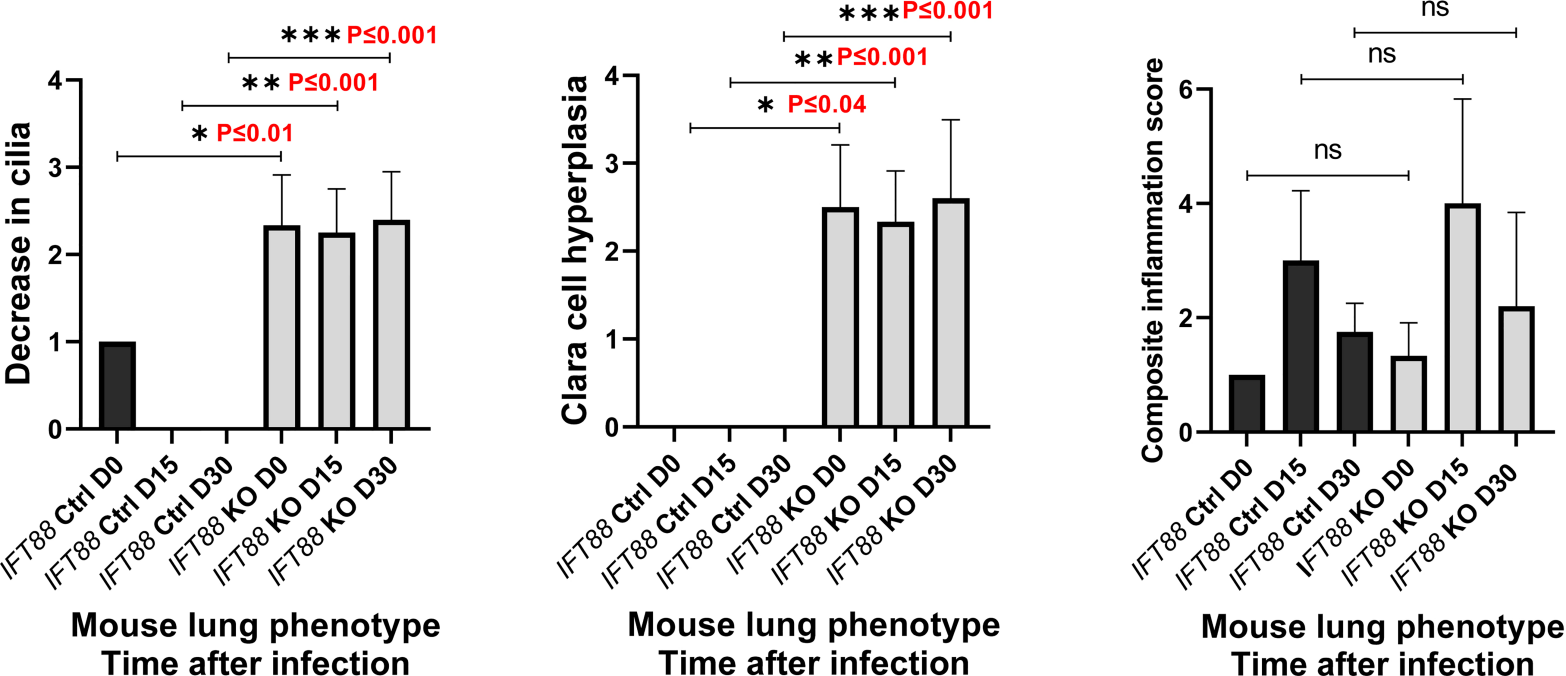
Lung histopathology was assessed in mice in which *IFT88* was deleted (*IFT* KO) compared to control mice (*IFT* Ctrl) at time of infection and at 15 and 30 days after infection with *Mabs* 390R. In this initial experiment with single cell *Mabs* thawed frozen stock, bacteria were cleared in the infected group by day 30. H & E stained slides of lung sections were examined by a veterinary pathologist and were scored for the three parameters as follows: 0=none; 1=minimal; 2=mild, 3=moderate; 4=marked. Values of 1 or less are likely within normal limits. The composite inflammation score was the sum of perivascular/peribronchiolar and parenchymal inflammation. Mean and standard deviation of 3 – 5 assessments in each group are shown along with significant differences. Decrease in cilia - *P ≤ 0.01 comparing *IFT88* control mice to *IFT88* KO mice at day 0; **P ≤ 0.001 comparing *IFT88* control mice to *IFT88* KO mice at day 15; ***P ≤ 0.001 comparing *IFT88* control mice to *IFT88* KO mice at day 30. Clara cell hyperplasia - *P ≤ 0.04 comparing *IFT88* control mice to *IFT88* KO mice at day 0; **P ≤ 0.001 comparing *IFT88* control mice to *IFT88* KO mice at day 15; ***P ≤ 0.001 comparing *IFT88* control mice to *IFT88* KO mice at day 30 (two-tailed *t*-test for unpaired samples). ns, not significant.

**Figure 2:**
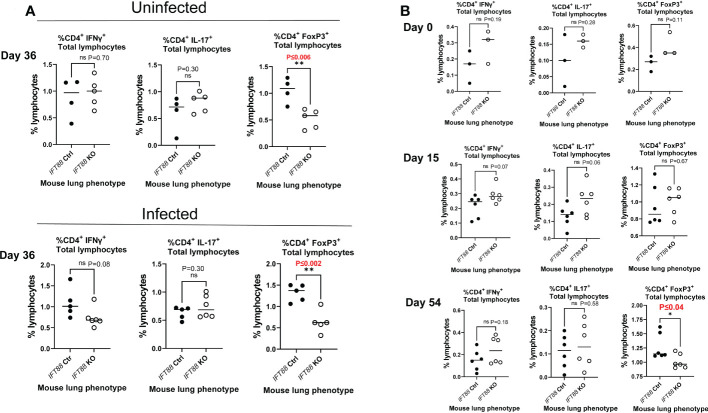
T cell subset analysis of cells isolated from mouse lung. **(A)** Percentage of T cells subsets from *IFT88* control (Ctrl) and *IFT88* KO mice that were uninfected or infected with thawed single cell suspension of *Mabs* from frozen stock in which persistent lung infection was not established. In both experiments, there was a significant decrease in the percentage of CD4^+^FoxP3^+^ in the *IFT88* KO group compared to the *IFT88* Ctrl group. Individual data points from 4-6 mice per group are shown with median values. Cells were 90-95% viable by trypan blue exclusion. There was a statistically significant difference comparing CD4^+^FoxP3^+^ T lymphocytes comparing *IFT88* control mice to *IFT88* KO mice in both uninfected and infected mice at day 36, 90 days after mice received tamoxifen; *P ≤ 0.006 uninfected mice, *P ≤ 0.002 infected mice (two-tailed *t*-test for unpaired samples). **(B)** Percentage of T cell subsets from *IFT88* Ctrl and *IFT88* KO mice infected with a low dose agarose bead inoculum of *Mabs* in which persistent infection was established out to the end of the experiment at 54 days. Individual data points from 3-6 mice per group are shown with median values. Cells were >95% viable by trypan blue exclusion. There was a statistically significant decrease in the percentage of CD4^+^FoxP3^+^ in the *IFT88* KO group compared to the *IFT88* control group at day 54, 107 days after mice received tamoxifen; P ≤ 0.04 (two-tailed *t*-test for unpaired samples). ns, not significant.

**Figure 3:**
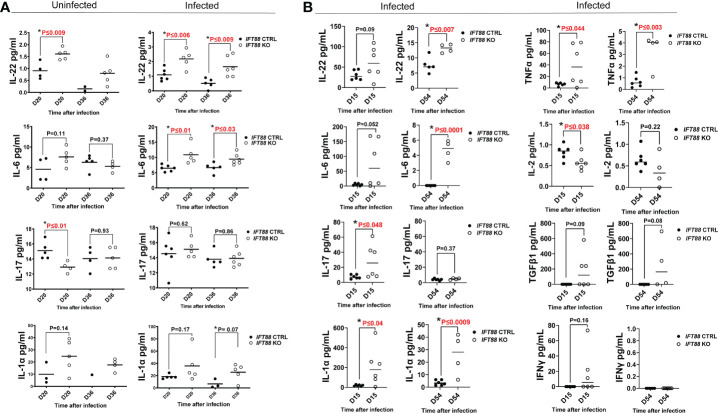
Cytokine analysis of BAL fluid from mouse lung. **(A)** Cytokine measurements from mouse lung BAL fluid from *IFT88* control (Ctrl) and *IFT88* KO mice that were uninfected or infected with thawed single cell suspension of *Mabs* from frozen stock in which persistent lung infection was not established. Individual data points from 1-6 mice per group are shown with median values. Statistical analysis was not performed unless at least 3 data points were present in each comparison group. Asterisks indicate statistically significant differences with P values shown (two-tailed *t*-test for unpaired samples). **(B)** Cytokine measurements from mouse BAL fluid from *IFT88* Ctrl and *IFT88* KO mice that were infected with *Mabs* using an agarose bead inoculum in which persistent infection was established out to the end of the experiment at day 54. Individual data points from 4-6 mice per group are shown with median values. Statistical analysis was not performed unless at least 3 data points were present in each comparison group. Asterisks indicate statistically significant differences with P values shown (two-tailed *t*-test for unpaired samples. ns, not significant.

In the earliest reported study of *Mabs* lung infection in mice, which we carried out (5), SCID mice that received a low inoculum of *Mabs* 390R single cell suspension intranasally developed persistent lung infection for 28 days, the duration of the experiment. We used this as our initial approach in this study with *Cre recombinase* positive/*IFT88* floxed mice (experimental group; *IFT88* KO) and *Cre recombinase* negative/*IFT88* floxed mice (control group; *IFT88* Ctrl). Our first experiment with intranasal inoculation of *Mabs* 390R single cell suspension prepared from frozen working stock did not result in persistent lung infection. At 15 days after infection, we recovered roughly the same number of bacteria that was in the original inoculum in 70% of both control and *IFT88* KO mice. However, the remaining animals at Day 15 had cleared the lung infection, and no bacteria was recovered from either group at 30 days (data not shown). As part of this experiment, a veterinary pathologist assessed lung histopathology in control and *IFT88* KO mice. *IFT88* KO mice demonstrated loss of cilia, Clara cell hyperplasia indicative of lung remodeling, and an increased composite inflammation score compared to control mice (**Figure 1**). We repeated this experiment using a low intranasal inoculum of *Mabs* 390R resulting in total lung deposition of 2.3 x 10^3^ total lung CFU (data not shown). At day 20 after infection, we only recovered between 50 and 250 total lung CFU from both the *IFT88* KO and control mice, and total lung CFU in all mice were below the limit of detection by day 36 after infection. Parallel groups of uninfected mice were included in this experiment for analysis of total lung CFU, BAL fluid cytokine analysis and total lung digests for analysis of T cell subpopulations. T cell subset analysis and cytokine levels from this experiment (**Figures 2A** and **3A**) are included with data from a separate experiment described below.

**Figure 4:**
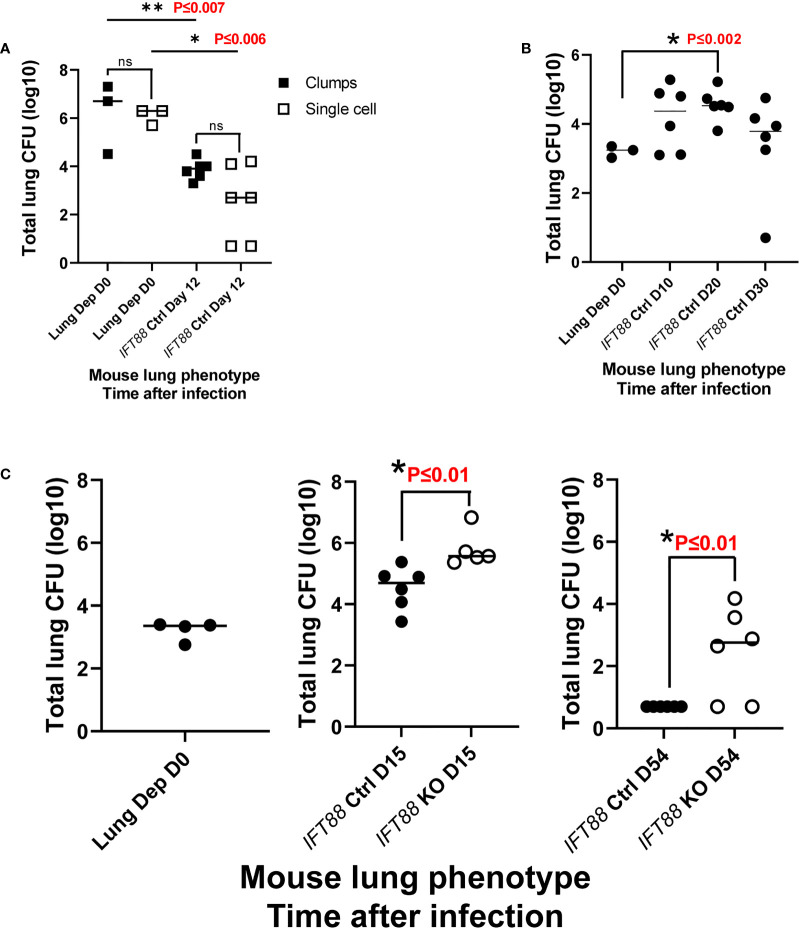
**(A)** Comparison of clump versus single cell *Mabs* lung inoculum in *IFT88* control (Ctrl) mice. *Mabs* 390R CFU were significantly decreased in the lungs of mice at day 12 with both clumped and single cell inocula. Individual data points from 3-6 mice per group are shown with median values. are shown. There were no differences in lung CFU comparing the different bacterial preparations at day 0 or day 12, however, there was a significant decrease in lung CFU from day 0 to day 12 for both lung inocula; **P ≤ 0.007 day 0 clumps compared to day 12 clumps; *P ≤ 0.006 day 0 single cell compared to day 12 single cell (two-tailed *t*-test for unpaired samples). **(B)** Pilot study with *Mabs* agarose bead inoculum using *IFT88* Ctrl mice. Individual data points from 3-6 mice per group are shown with median values. Lung deposition data represents day 0. Sustained productive infection was achieved in *IFT88* Ctrl mice using a relatively low agarose bead lung inoculum out to day 30 although lung CFU start to decline at day 30. There was a statistically significant increase in lung CFU at day 20 compared to day 0; *P ≤ 0.002 day 0 lung CFU compared to day 20 lung CFU (two-tailed *t*-test for unpaired samples). **(C)** Comparison of *Mabs* growth in lungs of *IFT88* KO vs *IFT88* Ctrl mice using agarose bead inoculum. *Mabs* persists in the lungs of *IFT88* KO mice throughout a 54 day course of infection and is cleared from the lungs of control mice. Individual data points from 4-6 mice per group are shown with median values. *Mabs* CFU inoculated into the lung were 2,000 CFU (3.3 log 10). Lung deposition data represents D0. There was a statistically significant difference in lung CFU comparing *IFT88* control mice to *IFT88* KO mice at day 15, *P ≤ 0.01 and day 54, *P ≤ 0.01 (two-tailed *t*-test for unpaired samples). At day 54 all values in the *IFT88* Ctrl mice were below the limit of detection. ns, not significant.

We hypothesized that aggregates of bacteria more closely mimic natural infection in which virulent *Mabs* cording variants (390R in our study) replicate as cords and form clumps (5, 15, 22, 23) and therefore might be more likely to induce persistent infection. To test this hypothesis, we used an overnight culture, which contained a mixture of individual bacteria and aggregates of various sizes, to infect mice. Despite achieving a high lung deposition, total lung CFU dropped 2-4 logs by day 12 with no significant difference compared to single cell suspension which had been prepared at the same time (**Figure 4A**). We next assessed *Mabs* growth in control mice using an agarose bead inoculum which has been reported to produce persistent *Pseudomonas aeruginosa* lung infection, and more recently, *Mabs* lung infection in mice with normal lung airways (10, 24, 25). A pilot experiment with a low *Mabs* agarose bead inoculum (average lung depositon of 1.7 x 10^3^ CFU) in *IFT88* Ctrl mice showed persistent lung infection out to 30 days, the duration of the experiment (**Figure 4B**). Thus, in our next experiment using this *Mabs* agarose bead inoculum, we compared growth of *Mabs* in the lungs of control mice to growth in the lungs of *IFT88* KO mice carried out to 54 days (approximately 8 weeks) post-infection (**Figure 4C**). Similar to **Figure 4B**, *Mabs* lung CFU increased in control mice at day 15, but fell below the limit of detection by Day 54. In contrast, 4 of 6 *IFT88* KO mice had significant numbers of CFU detected in the lungs at day 54 (**Figure 4C**). Importantly, the difference in *Mabs* lung CFU between control mice and *IFT88* KO mice in this model provides the basis for exploration of how abnormal lung airways influence the growth of *Mabs* in the lung. A key study, that formed the basis for our *Mabs* agarose bead inoculum (25), compared mouse BAL lung cytokines and inflammatory cells from mice receiving lung inocula of *P. aeruginosa* alone, *P. aeruginosa* embedded in agarose beads, agarose beads alone, or no treatment. The investigators reported that mice receiving *P. aeruginosa* either alone or embedded in agarose beads showed elevated BAL cytokines and increased BAL inflammatory cells compared to mice receiving no treatment. Importantly, mice receiving agarose beads alone did not differ from mice receiving no treatment in terms of cytokines or inflammatory cells. Thus, agarose is an immunologically inert component of the inoculum compared to the bacterial organism.

### The percentage of CD4^+^FoxP3^+^ T cells in the total lymphocyte population is decreased in the lungs of *IFT88* KO mice relative to control mice

We also analyzed T cell subset in the lungs and cytokines in the BAL fluid in this experiment, and compared the results (**Figures 2B** and **3B**) against the results from our earlier experiment in which we did not establish persistent infection with an intranasal inoculum of single cell suspension (**Figures 2A** and **3A**). T cell subset analysis showed a statistically significant decrease in the percentage of CD4^+^FoxP3^+^ T cells in the total lung lymphocyte population of *IFT88* KO mice compared to control mice in both uninfected and infected mice at D36 (90 days after receiving tamoxifen)(**Figure 2A**), and at D54 (107 days after receiving tamoxifen) using the agarose bead inoculum (**Figure 2B**). The significantly decreased percentage of T regulatory cells in the total lung lymphocyte population of *IFT88* KO mice indicates a shift toward a proinflammatory lung milieu compared to *IFT88* control mice. Furthermore, the decreased percentage of CD4^+^FoxP3^+^ T cells in the total lung lymphocyte population of *IFT88* KO mice is not dependent on infection with *Mabs* and is apparent 3 months after deletion of the *IFT88* gene (treatment with tamoxifen).

### *IFT88* KO mice have increased levels of lung proinflammatory cytokines compared to contrl mice.

*IFT88* KO mice infected using the agarose bead inoculum in which persistent infection was established (**Figure 3B**) showed much higher absolute levels of proinflammatory cytokines compared to mice inoculated with single cell suspension in which persistent infection was not established (**Figure 3A**). Levels were highest at day 15 with return toward baseline at day 54, although in some cases differences between *IFT88* KO and *IFT88* Ctrl mice persisted at this later time point (**Figure 3B**). These increased levels likely reflect the persistent *Mabs* infection established using the agarose bead inoculum. Overall, these results highlight the heightened proinflammatory response demonstrated in *IFT88* KO mice.

Of the proinflammatory cytokines assayed, IL-22 was noteworthy in that it was significantly elevated in *IFT88* KO mice relative to *IFT88* control mice in both the uninfected and *Mabs*-infected groups (**Figures 3A, B**), suggesting an important role for this cytokine in our infection model. Also noteworthy is the significant elevation of IL-6 in the lungs of infected *IFT88* KO mice relative to infected control mice independent of the establishment of persistent infection, supporting the inherent proinflammatory phenotype of *IFT88* KO mice (**Figures 3A, B**). In addition to IL-22, IL-6 may thus play a central in the pathogenesis of persistent infection in *IFT88* KO mice. IL-17 was not significantly elevated in *IFT88* KO mice compared to *IFT88* control mice in either uninfected or infected mice in the absence of persistent infection (**Figure 3A**). In contrast, it was significantly elevated at day 15 in infected *IFT88* KO mice compared to *IFT88* Ctrl mice where persistent infection was established using the agarose bead inoculum at day 15 (**Figure 3B**). T cell subset analysis of CD4^+^IL-17^+^ T cells at this time point shows an increased percentage of these cells at day 15 although this difference did not reach statistical significance (**Figure 2B**). This suggests that the elevated level of IL-17 at this time point represents a secondary, exaggerated proinflammatory response in *IFT88* KO mice that may have a direct bearing on the outcome leading to persistent infection at day 54.

Cytokines were assayed in both experiments, and in some cases additional cytokines were added for the experiment using the agarose bead inoculum (**Figure 3B**). Where specific cytokines were assayed in both experiments, data may be reported for only one experiment since values were below the detectable range of standard curves generated in the Luminex assay. This was the true for cytokines TNFα, IFNγ, and IL-2. Elevated levels of the proinflammatory cytokine TNFα were observed in infected *IFT88* KO mice relative to control mice (**Figure 3B**) in keeping with the proinflammatory phenotype of these mice. The lack of detectable IFNγ in *IFT88* Ctrl mice at days 15 and 54, as well as the non-significant transient elevation of IFNγ seen in at day 15 in *IFT88* KO mice (**Figure 3B**) suggests that in this model the Th1 immune response is not playing a role in pathogenesis. This is in keeping with our T cell subset analysis. Finally, IL-2 was significantly elevated in infected *IFT88* Ctrl mice compared to *IFT88* KO mice at day 15 (**Figure 3B**). This finding is consistent with the role that IL-2 is known to play in expanding the population of CD4^+^FoxP3^+^ T cells, and is reflected in our T cells subset analysis in this experiment at day 54.

### The lungs of *Mabs*-infected *IFT88* KO mice show an increased inflammatory response and greater numbers of granulomas compared to the lungs of infected control mice

**Figure 5:**
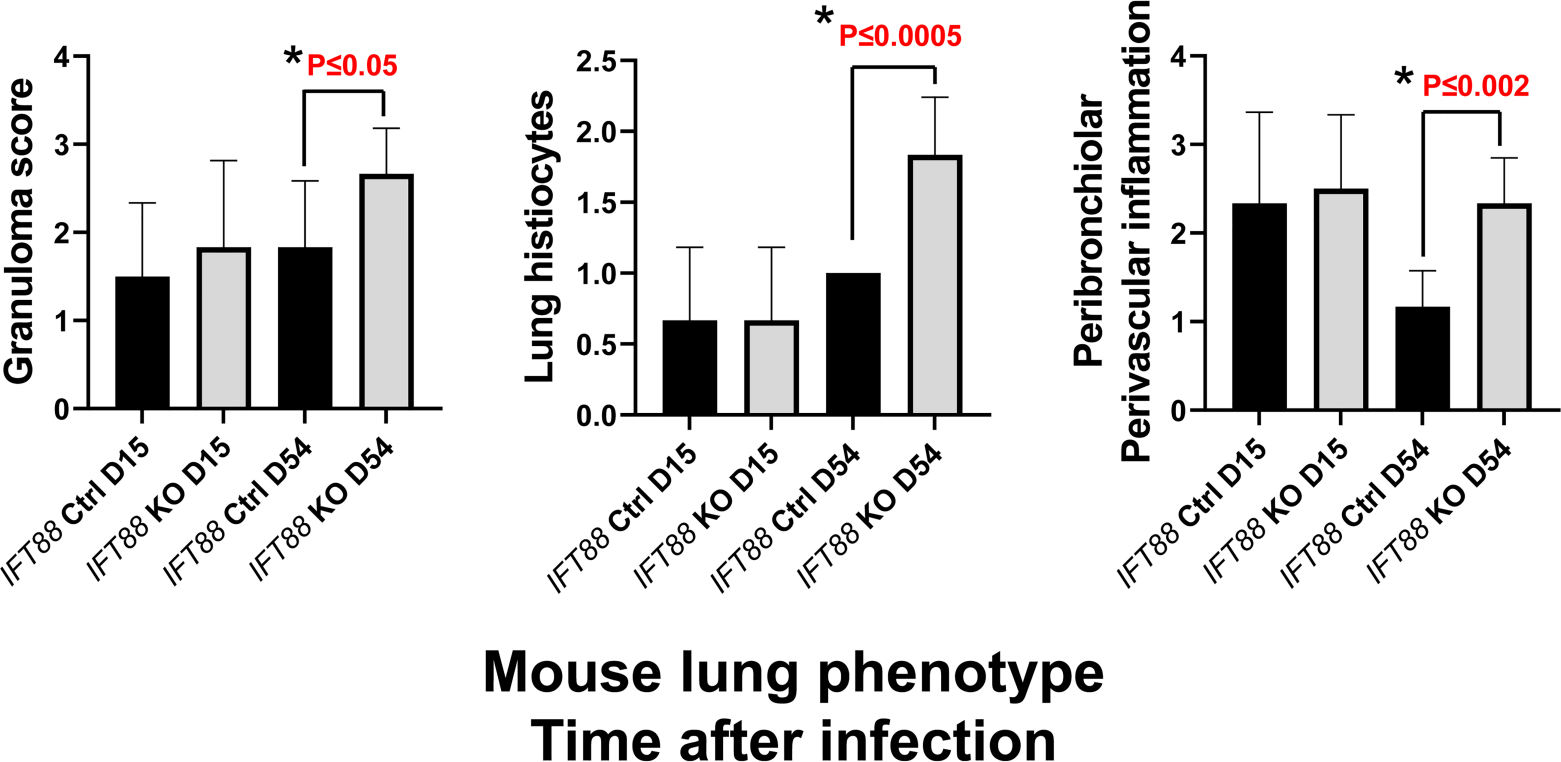
Lung histopathology was assessed in *IFT88* control (Ctrl) and *IFT88* KO mice infected with an agarose bead inoculum of *Mabs* in which persistent lung infection was established. H & E stained slides with lung sections were examined by a veterinary pathologist and graded on a scale of 0 (none), 1 (small number), 2 (moderate number), to 3 (large number) for the presence of granulomas, and a scale of 0 (none), 1 (mild), 2 (moderate) to 3 (marked) for perbronchiolar/perivascular inflammation and histiocytosis. Mean and standard deviation of values from 6 mice in each group are shown. Asterisks indicate significant P values comparing *IFT88* control mice (CTRL) to *IFT88* KO mice at 54 days (two-tailed *t*-test for unpaired samples).

**Figure 6:**
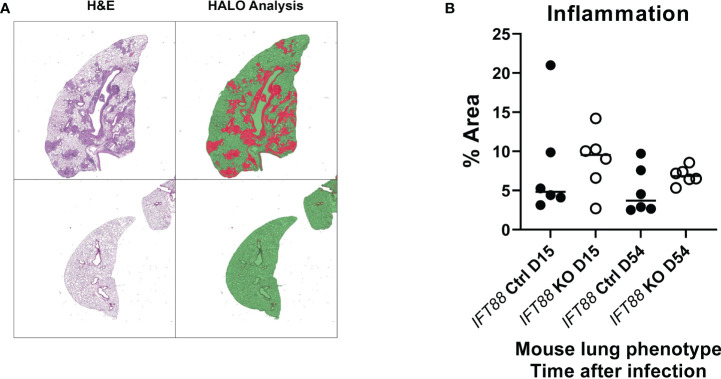
Lung airway inflammation was assessed using the HALO morphometry system in *IFT88* control (Ctrl) and *IFT88* KO mice infected with an agarose bead inoculum of *Mabs* in which persistent lung infection was established. **(A)** Recognition of areas of lung inflammation assessed by H & E staining closely correlated with areas identified by HALO morphometry. Lung parenchyma is identified as green areas without inflammation, and red areas as inflammatory cell infiltration. In **(B)**, the percentage of the total of lung area with inflammatory cell infiltrates was determined and compared between *IFT88* Ctrl and *IFT88* KO mice (n=6 mice per group at day 15 and day 54) with median values shown. Although median values were higher in *IFT88* KO group compared to the control group, the differences were not statistically significant (two-tailed *t*-test for unpaired samples).

In the experiment using the agarose bead inoculum, H&E stained lung sections examined by a veterinary pathologist were graded for the presence of granulomas, histiocytes in the alveoli, and peribronchiolar/perivascular inflammation. Consistent with *Mabs* persistence in *IFT88* KO mice, and the findings of our T cell subset analysis and BAL cytokine determinations, there was a statistically significant increase in all three parameters in *IFT88* KO mice compared to *IFT88* Ctrl mice at D54 (**Figure 5**). We also scanned slides using the HALO morphometry system and found increased inflammatory cells infiltrates in the lungs of *IFT88* KO mice compared to *IFT88* control mice (**Figure 6**), although these differences were not statistically significant. There was considerable variation in the extent of inflammation both among different lung lobes in a single mouse and among different mice within the same treatment group likely due to the low infecting inoculum and low level of bacterial persistence in this study. This variability is likely the reason that the differences did not reach statistical significance.

### Lung airway area and perimeter are not significantly increased in the lungs of *Mabs*-infected *IFT88* KO mice compared to the lungs of *Mabs*-infected control mice

**Figure 7:**
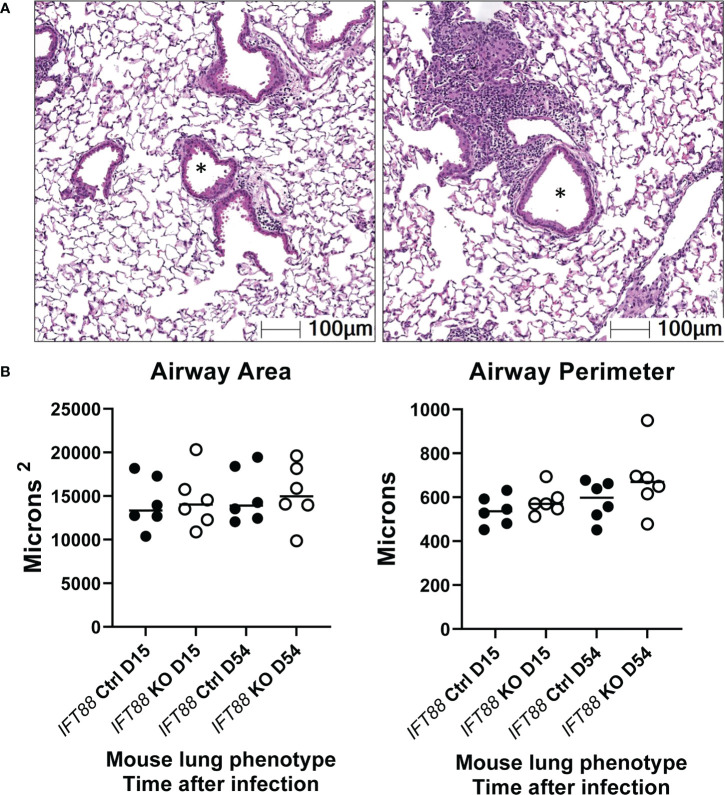
Lung airway size was assessed using the HALO morphometry system. In **(A)**, asterisks from two H & E lung sections illustrate typical airway lumina analyzed for measurements of airway perimeter and area.In **(B)**, a total of 15 airway cross-sections at the level of the terminal bronchioles from the lungs of each of 6 *Mabs*-infected mice per group at 15 and 54 days were compared. Both the area of the airway lumen in square microns and the perimeter of the airway in microns were measured with median values in each group shown. Although for both 15 and 54-day time points, median airway area and perimeter were slightly increased in *IFT88* KO mice compared to *IFT88* control (Ctrl) mice, the differences were not statistically significant when analyzed using a two-tailed t test.

As part of this assessment, slides were scanned for measurement of lung airway perimeter and area. Although both airway area and airway perimeter were increased in *IFT88* KO mice compared to *IFT88* Ctrl mice, these differences did not reach statistical significance (**Figure 7**). When analyzing airway area, we were not able to replicate the method of measurement previously described for these mice (12). It was not clear how the investigators determined the diameter of arteries and airways that often have highly irregular shapes.

## Discussion

*Mabs* is an emerging pathogen. In the United States, *MAC* is the most common NTM clinical pulmonary isolate followed by *Mabs* (26). The prevalence of *Mabs* lung infection is increasing in the general population where it has a predilection for individuals with bronchiectasis and COPD (27). Patients with bronchiectasis are prone to lung airway colonization with NTM (2). In spite of being one of the most virulent NTM, *Mabs* does not cause systemic or invasive parenchymal lung infection in patients with normal lung airways unless they have severe defects in cell-mediated immunity. What is not known is why, after a period of colonization of abnormal lung airways, progressive lung infection occurs despite the fact that most patients with bronchiectasis have seemingly intact cell-mediated immune responses.

Establishing *Mabs* lung infection in mouse models has been problematic. We were the first to demonstrate that *Mabs* could persist in the lungs of mice, specifically immunocompromised SCID mice (5). All our studies have used a low infecting inoculum to mimic human lung infection in which small numbers of *Mabs* are felt to gain access to abnormal lung airways after microaspiration of *Mabs* colonizing the oropharynx (5, 15, 28). In a *Mabs* lung infection model utilizing GM-CSF knockout mice, initial infection was with a high inoculum of a predominantly smooth *Mabs* variant. *Mabs* smooth variants express glycopeptidolipid and form biofilms (15, 16). With spontaneous loss of GPL, *Mabs* becomes a cording, rough colony phenotype that is invasive, virulent and capable of inducing an inflammatory host immune response (17, 18). Mice in which GM-CSF has been deleted have defects in macrophage differentiation along with abnormal lung surfactant accumulation which could contribute to the ability of the smooth *Mabs* variant to persist in the lungs of these mice, form biofilm, and result in the emergence of the invasive rough *Mabs* variant (15, 28, 29). In this GM-CSF knock out mouse model, lung CFU declined over the first month after infection but then began to increase out to 4 months post-infection, the duration of the experiment. At the later time points when lung CFU were increasing, primarily *Mabs* rough variants were recovered from the lung and histopathologic findings were consistent with the development of bronchiectasis (9). Whether persistent infection in this model was secondary to development of bronchiectasis or the lack of GM-CSF is an important question since the results of our study support the concept that abnormalities in lung airways such as loss of cilia are a pre-condition for establishment of *Mabs* infection which may not be contingent on the presence of bronchiectasis (30).

We have used a previously described Cre/*loxP* mouse model in which deletion of the *IFT88* gene, which encodes for the protein Polaris, reportedly leads to development of bronchiectasis (12). This study showed that *IFT88* deletion resulted in loss of cilia, airway remodeling, and had no effect on mucus airway production. With *IFT88* deletion, we observed loss of airway cilia in mice, but were not able to replicate significantly increased airway diameter reported in this study using a different methodology for lung airway measurement. We measured airway perimeter and diameter in the mice approximately 4 months after *IFT88* deletion using HALO scanning microscopy, and although median values were higher in mice in which *IFT88* had been deleted, these differences were not statistically significant. Importantly, we demonstrate *Mabs* persistence in the lungs of mice with *IFT88* deletion for at least 8 weeks after infection following a low dose infecting inoculum (approximately 3-4 months after deletion of *IFT88*). In contrast, by 8 weeks, *Mabs* was below the limit of detection in the lungs of control mice. The small differences in airway size with *IFT88* deletion that we observed in our experiments suggests that factors other than altered mucociliary clearance and/or enlarged airways are involved in *Mabs* persistence in *IFT88* KO mice. As discussed below, *Mabs* persistence in the lungs of *IFT88* KO mice may be due to the altered immunologic milieu we found in these mice.

In our study, we found that that we could not establish persistent infection with single cell bacterial suspensions from frozen stock or from overnight broth grown cultures. We found that a low dose *Mabs* agarose bead inoculum based on prior studies with *Pseudomonas aeruginosa* (24) could establish prolonged infection in our control mice over the course of 30 days. During the time our studies were being conducted, there was a report that showed establishment of *Mabs* lung infection using a high *Mabs* agar bead lung inoculum in immunocompetent C57BL/6NCrl mice (10). The prolonged persistence of *Mabs* in the lungs of mice with normal airways in this study may be due to the size of the infecting inoculum and/or strain differences compared to the mice we used in our study. Our infecting inoculum was approximately 1 X 10^4^ CFU in our two agarose bead inoculum experiments versus 1 X 10^5^ CFU in the prior study. In addition, we used nonsurgical inoculation into the trachea compared to dissection of the trachea with direct *Mabs* deposition in the prior study. A direct comparison is not possible, as lung deposition immediately following lung inoculation was not reported in the prior study (10). As stated, we aimed for low lung deposition of *Mabs* agarose beads in our assay to bring out differences between our control and *IFT88* KO mice. In natural *Mabs* infection in humans it is presumed that biofilm formation by colonizing smooth variants is present at the time rough invasive variants spontaneously arise in the lung (28). The presence of lung airway biofilm may be a prerequisite for persistent lung infection to occur. We speculate that the provision of agarose in the infecting inoculum may provide de facto biofilm along with the infecting bacteria. The utility of our model is that we have established experimental and control conditions in the same mice that will allow for elucidation of factors that facilitate *Mabs* persistence in the lung associated with lung airway ciliopathy. This model may also have relevance for other NTM lung pathogens such as *MAC*.

The data from our study indicate that deletion of *IFT88* in mice leads to development of a pro-inflammatory lung phenotype with elevation of proinflammatory cytokines and a reduced percentage of anti-inflammatory T regulatory cells. The cytokine IL-22 was the only cytokine elevated in both uninfected and infected *IFT88 KO* mice in both experiments. IL-22 is a pleiotropic cytokine with multiple functions determined by the local milieu at the site of its release. In the absence of other cytokines, it functions to repair and maintain the integrity of respiratory epithelium (31). This would explain the elevated levels we found in uninfected *IFT88* KO mice that are undergoing airway remodeling due to deletion of *IFT88*. In addition, in all *IFT88* KO mice whether infected or not, the percentage of CD4^+^FoxP3^+^ T cells was decreased relative to *IFT88* control mice. This was observed at 3-4 months post deletion of *IFT88*. There is some data linking IL-22 with decreased CD4^+^FoxP3^+^ T cells although the exact mechanism for these observations is unclear since T cells do not express IL-22 receptor (32, 33). Our model affords an opportunity for further investigation of this association.

In contrast to its regenerative effect on airway epithelium in the absence of other cytokines, IL-22 acts with IL-17 to promote lung airway inflammation (31). In our agarose bead inoculum experiment in which *Mabs* persisted in the lungs of *IFT88* KO mice, we found an increased percentage of CD4^+^IL-17^+^ T cells in the lungs of *IFT88* KO mice compared to control mice at D15 along with a significant increase in the level of IL-17 in the lungs of these mice at this time point. These results suggest that the cytokine milieu at this early time point after *Mabs* infection creates proinflammatory conditions that promote *Mabs* persistence that is then facilitated by the relative decrease in CD4^+^FoxP3^+^ T cells observed at day 54.

One consistent finding in both experiments in which we measured cytokine BAL levels was elevation of IL-6 in *IFT88* KO mice compared to control mice. The relevance of our finding to human disease is demonstrated by the finding that IL-6 is elevated in BAL fluid from the lungs of humans not colonized with bacteria who have non-cystic fibrosis (non-CF) bronchiectasis compared to healthy controls (34). Another study found that IL-6 levels were elevated in the sputum from children with non-CF bronchiectasis compared to sputum from children with CF and healthy adult controls (35). Low levels of IL-6 in sputum from subjects with CF in this study (35) may be the result of degradation, and/or inhibition of release of IL-6 in the lungs of CF patients who are colonized with *Pseudomonas aeruginosa* (36). The functions of IL-6 include promoting differentiation of naive CD4^+^ T cells. IL-6 in combination with TGF-β promotes Th17 differentiation from CD4^+^ T cells, and IL-6 inhibits TGF-β induced T regulatory cell differentiation (37). This is consistent with our observations and may in part account for the decrease in the percentage of T regulatory cells in the lung lymphocyte population we observed in our *IFT88* KO mice compared to control mice.

It is noteworthy that in the experiment using the agarose bead inoculum in which we measured IL-2, there was a significant increase in IL-2 in the BAL fluid of *IFT88* control mice compared to *IFT88* KO mice at D15. Among its many functions, IL-2 plays a critical role in maintenance and expansion of Treg cells (38), consistent with our finding that T regulatory cells are decreased in *IFT88* KO mice relative to *IFT88* Ctrl mice at the subsequent D54 time point.

The other notable findings in our agarose bead inoculum experiment were the significantly elevated levels of IL-1α and TNFα in *IFT88* KO mice relative to control mice at day 15, with the difference still present and significant at day 54 after infection. IL-1α is a proinflammatory cytokine that synergizes with TNFα, promotes release of IL6, and is elevated in structural lung disease (39), all of which are consistent with our model and our observations. One of the important functions of TNFα is to promote granuloma formation (40). Our histopathology assessment in this experiment showed increased granuloma formation in *IFT88* KO mice compared to control mice at day 54. At this time point, *Mabs* persisted in the lungs of *IFT88* KO mice compared to control mice. Elimination of pathogens from granulomas requires the presence of macrophage-activating cytokines such as IFNγ. In this experiment, several *IFT88* KO mice had increased levels of IFNγ at D15; however, levels were not detectable at D54. There was no detectable IFNγ in the lungs of control mice at either time point in spite of *Mabs* clearance by D54. These results suggest that CD4^+^IFNγ^+^ - mediated responses are not playing a role in clearance of *Mabs* infection from either group of mice in our experiments.

An important question raised by our results is how the reduced percentage of T regulatory cells in the total lung lymphocyte population, and the increase in proinflammatory cytokines we have observed in the lungs of mice with ciliopathy due to loss of *IFT88* relates to increased growth of *Mabs*? One possible explanation is that recruitment of cells such as monocytes *via* the inflammatory response provides conditions conducive to growth of *Mabs*. Nonactivated monocyte/macrophages are a permissive substrate since *Mabs* is an intracellular pathogen and our data indicate that macrophage-activating Th1 responses in our model do not appear be involved in clearance of *Mabs* from our control mice. Evidence suggests that dysregulation of T regulatory/Th17 balance with an inadequate T regulatory response favors *Mycobacterium tuberculosis* by promoting ongoing recruitment of inflammatory cells to granulomas which are ineffective at containing the organism (41). The concept that nonactivated monocyte-derived macrophages, newly recruited to the site of infection caused by an intracellular pathogen, provide the substrate for pathogen growth is also supported in other infection models (42). It is noteworthy that histopathologic assessment showed a significant increase in granulomas and lung histiocytes at D54 in *IFT88* KO mice compared to control mice with *Mabs* persisting in the lungs at this time point. A recent study using the established zebrafish model of *Mabs* pathogenesis demonstrated that growth of the inflammatory *Mabs* rough variant (the morphotype used in our study) results from host TNFα-driven necrotic granulomas. Restriction of virulent *Mabs* rough variant growth is achieved by host T regulatory cells which associate with these granulomas (43). These results parallel our immunologic findings in which a reduced percentage of T regulatory cells in *IFT88* KO mice is associated with increased *Mabs* growth using a completely different model of *Mabs* infection. This supports the use of the *IFT88* KO mouse model to study the interplay between *Mabs* lung infection and host immune responses.

The initial objective of these experiments was to study the effect of bronchiectasis on the ability of *Mabs* to persist in the lungs of mice. However, although we observed increased lung airway area and perimeter measurements in *IFT88* KO mice compared to control mice, the differences were not statistically significant. Thus, the significant immunologic differences along with *Mabs* persistence in our experiments comparing *IFT88* KO mice to control mice suggest an effect that goes beyond abnormal mucociliary clearance due to loss of cilia. An emerging area of research is the environmental sensing and signaling functions of ciliary transport proteins independent of motility. A number of studies have provided evidence that sensory/signaling functions that have been described for nonmotile primary cilia in many tissue sites also occur in motile cilia (44). In addition, certain ciliary intraflagellar proteins play a conserved role in intracellular transport - specifically, in trafficking TCR/CD3 to the immune synapse of T cells (45). In relation to our study, *IFT88* regulates NFκB signal transduction in response to inflammatory signals in ciliated cells as well as in non-ciliated cells such as macrophages. For example, loss of *IFT88* reduces NFκB – mediated expression of *NOS2* in mice (46). Studies in humans have demonstrated dysregulation of cilia cell differentiation in airway epithelium of COPD patients which may be due to aberrant responses to TGFβ1, as well as aberrant localization of IFT88 in ciliated respiratory epithelial cells in patients with CF (47, 48). In addition, IFT proteins, including IFT88, have been demonstrated to play a role in autophagy with crosstalk occurring between primary cilia found on all cells, and cellular autophagic machinery (49). The fact that the process of autophagy is anti-inflammatory (50) could in part account for the proinflammatory lung phenotype we have found in *IFT88* KO mice.

In conclusion, we have demonstrated a novel mouse model in which a low infecting agarose bead lung inoculum of *Mabs* results in persistent infection in mice with lung airway ciliopathy. Although we initially chose this model due to the reported development of bronchiectasis in these mice, our results showed only a trend toward increased lung airway size, albeit using a different methodology for measurement. Importantly, we observed a marked proinflammatory lung phenotype in mice with lung airway ciliopathy associated with deletion of *IFT88*, along with a decreased percentage of CD4^+^FoxP3^+^ T lymphocytes out of the total lymphocyte population in the lungs of these mice compared to control mice. There is emerging evidence that *IFT88* encodes for a protein that has immunological functions as well as an essential role in normal cilia development. The clinically relevant question raised by our study is whether lung airway ciliopathy, either congenital or acquired because of bronchiectasis, alters the immunologic milieu in the lungs and contributes in the pathogenesis of *Mabs* lung infection, independent of associated abnormalities in mucociliary clearance.

## Data availability statement

The original contributions presented in the study are included in the article/**Supplementary Material**. Further inquiries can be directed to the corresponding author.

## Ethics statement

The animal study was reviewed and approved by Institutional Animal Care and Use Committee of the University of New Mexico Health Sciences Center.

## Author contributions

AN and AH conducted experiments and contributed to design of experiments. TW contributed to experimental design and interpretation of data. TB conceived the experiments, contributed to experimental design, interpreted the data and wrote the final version of the manuscript. All authors contributed to the article and approved the submitted version.

## Funding

This work was supported by National Institutes of Health grant AI137633 to TB. Donna F. Kusewitt, DVM, PhD provided histopathologic assessment of mouse lung with support provided by the UNM Comprehensive Cancer Center Support Grant NCI P30CA118100 and the Animal Models Shared Resource.

## Acknowledgments

We would like to thank Barbara Brown Elliot, MS, MT(ASCP)SM (UT Tyler Health Science Center) who determined the subspecies of *Mycobacterium abscessus* used in this study.

## Conflict of interest

The authors declare that the research was conducted in the absence of any commercial or financial relationships that could be construed as a potential conflict of interest.

## Publisher’s note

All claims expressed in this article are solely those of the authors and do not necessarily represent those of their affiliated organizations, or those of the publisher, the editors and the reviewers. Any product that may be evaluated in this article, or claim that may be made by its manufacturer, is not guaranteed or endorsed by the publisher.
