# Supplementary figures and images for: Mice with lung airway ciliopathy develop persistent Mycobacterium abscessus lung infection and have a proinflammatory lung phenotype associated with decreased T regulatory cells
Source: Front Immunol. 2022 Nov 25;13:1017540. doi: 10.3389/fimmu.2022.1017540 (PMC9732727; doi:10.3389/fimmu.2022.1017540)

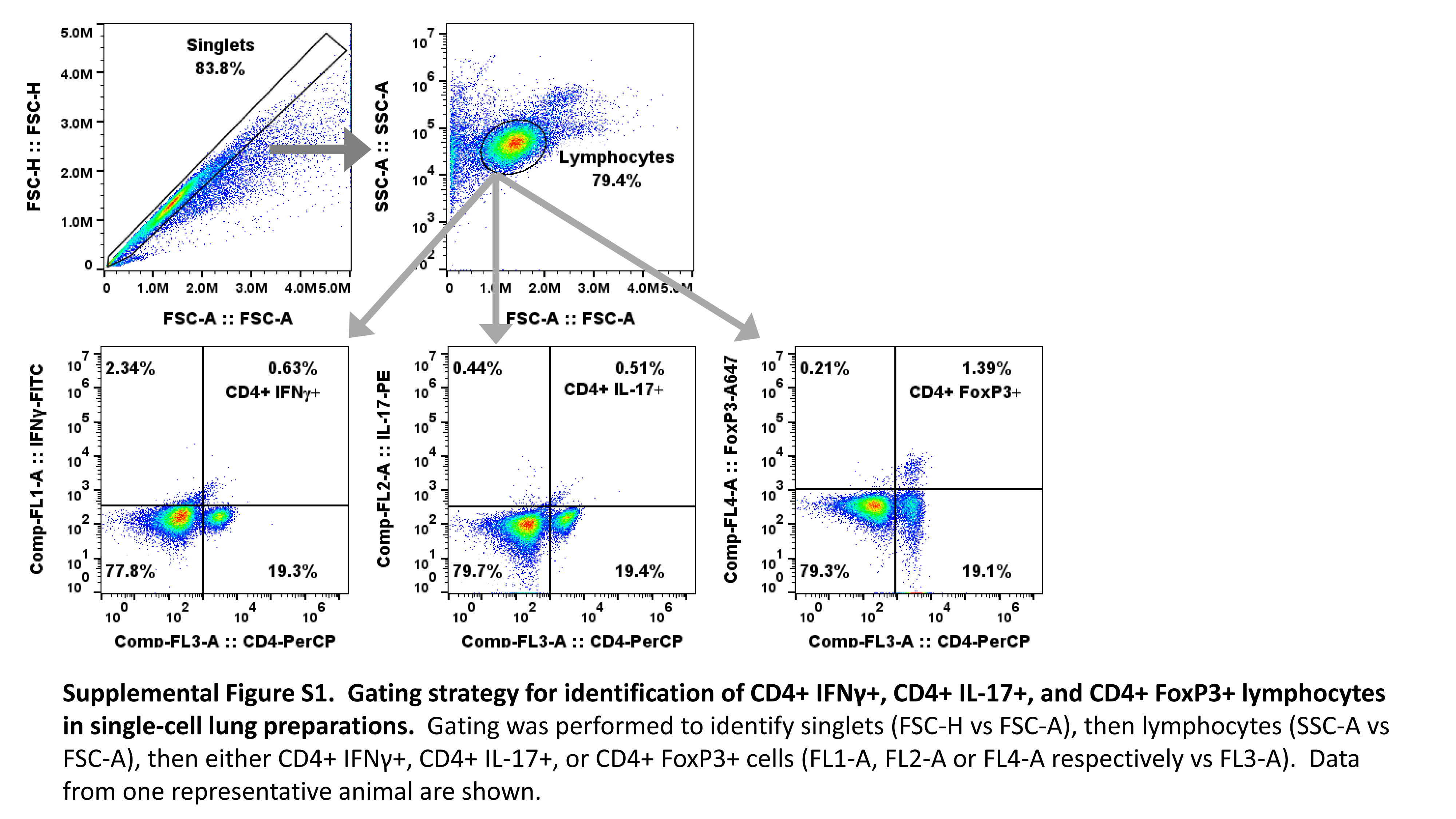

Supplement: Supplementary file 1 [file Image_1.jpeg]
